# Dr. Isaac N. Quimby

**Published:** 1898-06

**Authors:** 


DR. ISAAC N. QUIMBY, of Jersey City, died at his home May 6, 1898, in the sixty-seventh year of his age. He was a prominent actor in the affairs of the American medical association, where he was generally on his feet whenever there was a debate of a controversial character. His long hair and grizzly beard presented a striking appearance and almost everyone knew him who has ever attended the meetings of the association. He will be greatly missed among his colleagues and by his personal friends, who were many.

